# The equitable aging in health conceptual framework: international interventions infusing power and justice to address social isolation and loneliness among older adults

**DOI:** 10.3389/fpubh.2025.1426015

**Published:** 2025-03-10

**Authors:** Angela K. Perone, Leixuri Urrutia-Pujana, Leyi Zhou, Mo’e Yaisikana, Barbara Mendez Campos

**Affiliations:** ^1^School of Social Welfare, University of California, Berkeley, Berkeley, CA, United States; ^2^Faculty of Law, Universidad del País Vasco/Euskal Herriko Unibertsitatea (UPV/EHU), Bilbao, Spain; ^3^School of Social Work, Boston College, Chestnut Hill, MA, United States

**Keywords:** social determinants of health, intersectionality, power, justice, interventions

## Abstract

**Introduction:**

Social isolation and loneliness among older adults have garnered significant international attention, particularly as structures and services have evolved during a global pandemic. A growing body of research underscores disparities in social isolation and loneliness among intersecting social (e.g., race, ethnicity, age, gender, sexual orientation, disability) and physical (e.g., rural/urban) locations. While empirical data about these global trends has increased, conceptual and theoretical frameworks are underdeveloped about disparities in social isolation and loneliness, especially from a global perspective. This article presents a novel equitable aging framework to help contextualize, understand, and explain how power influences disparities in social isolation and loneliness among older adults.

**Equitable aging in health conceptual framework:**

Equitable aging builds on principles in critical gerontology, public health concepts of social and political determinants of health, international human rights, and intersectionality frameworks to present a new conceptual framework for researchers, policymakers, and practitioners. Equitable aging centers five domains of power (intrapersonal, interpersonal, disciplinary, structural, and cultural) as critical components (or hub) that drive six political and social determinants of health (economic stability, education access and quality, health care access and quality, neighborhood and built environment, social and community context, and laws and politics). The sixth determinant of health (laws and policies) incorporates international human rights (economic, social, cultural, civil, political rights). When justice is infused in these domains of power, political and social determinants of health can produce equitable aging outcomes. The Equitable Aging in Health Framework presents a new tool that incorporates justice and power to help understand and explain disparities in social isolation and loneliness and ultimately how to achieve equitable opportunities for social connections for older adults.

**Discussion:**

To illustrate the utility of this conceptual framework, this article presents six case studies of interventions in China, Taiwan, Spain, Sweden, Mexico, and the United States that employ this framework to address social isolation and loneliness among diverse communities of older adults. These interventions propose new services, programs, and policies that infuse different paradigms of justice and address domains of power in various ways to build social connections and support for older adults.

## Introduction

Social isolation and loneliness have become serious public health concerns as more research links them to poor health outcomes. For example, social isolation among older adults has been associated with increased risk of heart disease, stroke, and dementia, premature death that might exceed the impacts of smoking, obesity, and physical inactivity (1), worse oral health (2), and higher emergency room visits and medical costs (3). Loneliness among older adults has been associated with higher rates of depression, anxiety, and suicide (1), cognitive decline (4), and higher emergency room visits and medical costs (3). Evidence suggests that spousal support, social networks, adaptive organizational change, and a responsive public sector may help mitigate some of these effects (5).

While often interrelated, social isolation and loneliness are distinct concepts. Social isolation refers to an objective state of having a small network of relationships and thus limited interactions with others (6). Loneliness is a subjective feeling or “social pain” that arises from limited desired or actual social connections (4, 6).

Some older adults face elevated or unique risks associated with loneliness or social isolation, given their intersecting positionalities (e.g., race, ethnicity, age, gender, sex, sexual orientation, marital status, (dis)abilities, socio-economic status, etc.), which can produce complex outcomes. For example, one study found that for Black older adults, social disconnectedness is negatively associated with physical health while perceived isolation is negatively associated with mental health (7). Another study found that greater loneliness had a stronger effect on white older adults than for Black older adults, which the authors suggested may mean that loneliness is less of a direct mental health predictor for persons with fewer economic resources with greater needs for instrumental support (i.e., transportation, money for bills and groceries) (8). LGBTQIA+ older adults are more likely to live alone, be single and childless, and rely more on “families of choice” for social connections (9–12), which can impact experiences of social isolation and loneliness. One study found that unemployment is associated with higher levels of social isolation among transgender adults (13). Living in a rural location may also increase one’s risks of experiencing loneliness and social isolation [e.g., (14, 15)]. Evidence further suggests that those over the age of 75 or 80 have higher levels of loneliness compared to younger adults (16, 17).

Positionalities reflect one’s positions of power in relation to others in various social, political, and economic structures, cultural contexts, and interpersonal dynamics [e.g., (18–21)]. Positionalities are dynamic and can shift with changing contexts (22, 23). While the term positionality gained traction among critical scholars to identify the social location of researchers (20), it has since been applied beyond the research context to understand and interrogate complex social dynamics in a range of circumstances, including participatory budgeting (21), gender inclusion in esports organizations (24), refugee entrepreneurship (25), racial and ethnic disparities in school discipline (26), and a sense of belonging among older adults amidst socio-cultural neighborhood changes (27).

While positionalities likely have a significant impact on one’s experiences of social isolation and loneliness, few articles address power when examining these issues. Most research on social isolation and loneliness also focuses on experiences of older adults in the United States over any other country [e.g., (28–30)]. Moreover, conceptual and theoretical frameworks are underdeveloped in this body of literature, especially frameworks that incorporate power. This article, thus presents a novel equitable aging conceptual framework that builds on prior literature on social and political determinants of health and power to help contextualize, understand, and explain how power may influence disparities in social isolation and loneliness among older adults. The Equitable Aging in Health Conceptual Framework incorporates five domains of power that shape social and political determinants of health. By infusing justice into interventions for social isolation and loneliness, we argue that these interventions can better achieve equitable outcomes for older adults that reduce disparities for social isolation and loneliness.

This article begins by describing social and political determinants of health and domains of power. Next, it describes how it incorporates these concepts into the Equitable Aging in Health Conceptual Framework. It concludes with six international case examples based on the authors’ academic and professional experiences in these countries that illustrate application of this conceptual framework for urban community-dwelling older adults in China, rural Indigenous older adults in Taiwan, nursing home residents in Spain, non-European older migrants in Sweden, older adults in Mexico, and LGBTQIA+ older adults in the United States.

## Social and political determinants of health

Social determinants of health (SDOH) comprise the structural and intermediate contexts in which people are born, grow, work, live, and age as well as the systems and structures that impact the conditions of their daily lives (31).^1^ The concept arose, in part, as a response to a dominant medical model of health that explained health problems or disparities solely or predominantly as a function of individual behavior, lifestyle, or biology [e.g., (32–34)]. Structural social determinants of health focus on the historical, socioeconomic, political, and cultural factors that shape health (e.g., governing process, public policies) (31). Intermediate social determinants of health address conditions in one’s daily life that shape health (e.g., pay, working conditions) (31). Social determinants of health can be grouped into five categories: economic stability, education, physical environment (also referred to sometimes as the neighborhood and built environment), healthcare, and social and community context [e.g., (35)]. Inequities among social determinants of health drive health disparities and produce inequitable outcomes across the life course, including inequities that exacerbate social isolation and loneliness among older adults. As more policymakers, scholars, and organizations recognize loneliness and social isolation as public health issues, social determinants of health have increasingly been linked to these issues [e.g., (36)].

Daniel Dawes (37) argues that political determinants of health further shape social determinants of health, including inadequate transportation, food deserts, and higher pollution in some neighborhoods. He adds that public policies often produce social determinants of health inequity (37) and that policies are “the determinants of the determinants” [(37), p. 45]. For example, when a transportation policy removes a bus route that allows community members to access nutritious food from a local grocery store, that policy contributes to health inequity. Public policies can inversely also produce social determinants of health equity. For example, local policies that subsidize rideshares or public taxis, especially when public transportation is unavailable or inaccessible, can help ameliorate prior conditions of health inequity and produce more equitable outcomes for obtaining nutritious food. When community members come together and advocate for new transportation policies, they also elevate community consciousness about the range of possibilities for addressing root causes of health inequities, including transportation policies (37).

While the concept of political determinants of health has mostly been applied to domestic policy contexts in the United States [e.g., (37–39)], it has broad application in an international context [e.g., (40–42)] and, in fact, emerged earlier in a 2005 article on global health by Professor Ilona Kickbusch who argued that “the crisis in global health is not a crisis of disease, it is a crisis of governance” (p. 246) (137). Global policies can also shape social determinants of health inequity and equity, including global policies on international human rights. International human rights laws arose from the atrocities of two global wars and the subsequent codification of the Universal Declaration of Human Rights (43). The Universal Declaration of Human Rights outlines five main categories of human rights: economic, social, cultural, civil, and political rights (44). Human rights organizations and scholars have long recognized the connection between social determinants of health and human rights [e.g., (45–47)]. Human rights can be incorporated into any of the core categories identified as social determinants of health, as well as the broader framework of political determinants of health.

The COVID-19 pandemic sparked international conversations about whether or not health-imposed quarantines violate (or support) international human rights [e.g., (48, 49)], amidst increasing evidence of the negative health impacts of social isolation during the pandemic [e.g., (50–53)] and health disparities as to who was at risk of contracting and dying of COVID-19 [e.g., (13, 54–56, 136)]. The complexity embedded in policies that mandate quarantines during public health emergencies underscores the importance of power. For example, what is the scope of these quarantines? Why are quarantines imposed and under what circumstances are they justified? Who has the power to implement and enforce these quarantines and against whom? How should quarantines be designed and enforced? While power is implicitly embedded in political and social determinants of health, we argue that it should be explicitly named and considered when identifying possible public policies or community-based solutions to addressing health inequities, including inequities in social isolation and loneliness.

## Domains of power

Power shapes material realities and social relations (57), the production of knowledge (58), and perpetuates hegemonic conditions that facilitate dominance of one or more groups, ideas, values, or beliefs in society [(59); e.g., (139, 140)]. Patricia Hill Collins and Sirma Bilge (60) identify four domains of power: cultural, structural, disciplinary, and interpersonal. Cultural power encompasses the creation, perpetuation, and values attached to various meanings, interpretations, and ideologies (60, 61). Cultural power addresses *why* oppression occurs and societal justifications for inequities. Structural power includes the ways that social institutions are organized to reproduce subordination over the lives of particular people (61). Structural power addresses *what* drives oppression. Disciplinary power includes the ways that governments, bureaucracies, and other social actors regulate thought and behavior through subtle rules and practices, and social processes (61, 62). Disciplinary power addresses *how* oppression is enacted. Interpersonal power encompasses the everyday interactions among people (60). These domains of power are mutually constructed and thus do not exist in individual silos (60). Glover Reed et al. (63) also discuss an intrapersonal domain of power, which focuses on individual characteristics, attitudes, skills, and knowledge. Intrapersonal and interpersonal power addresses *who* enacts and internalizes oppression.

Experiences of social isolation and loneliness are shaped by these five domains of power. For example, in one study, researchers argue that rumors and misinformation during initial months of COVID-19 lockdown created moral panic that resulted in fear and social isolation for Muslims living in Nepal (64). Here, migration policies in Nepal and India as well as economic conditions that necessitated migration illuminate the presence of structural power. Cultural power intersected with structural power when rumors and mis/disinformation on social media portrayed Muslims as carriers and transmitters of COVID-19. Stereotypes of Muslims helped non-Muslims justify differential treatment toward Muslims in Nepal. While Muslims in Nepal were allowed to engage in social activities, the rumors and misinformation prompted many to disengage, in part, from concerns that others would invoke these rumors and misinformation to police their activities and behavior—illustrating how disciplinary power can impact social isolation. Interpersonal power emerged through interactions between Muslims and non-Muslims in Nepal that further reinforced beliefs that Muslims from India had COVID-19 and that they should self-isolate for 14 days. Internal stigma and shame stemming from these experiences further illustrate how intrapersonal power can shape social isolation. By understanding how power shapes experiences of social isolation and loneliness, researchers, practitioners, and policymakers can create interventions that address the diverse needs of older adults at multiple levels (from the individual to the structural). The Equitable Aging in Health Conceptual Framework presents a tool that foregrounds power to help render it more visible for public health interventions.

## The Equitable Aging in Health Conceptual Framework

**Figure 1:**
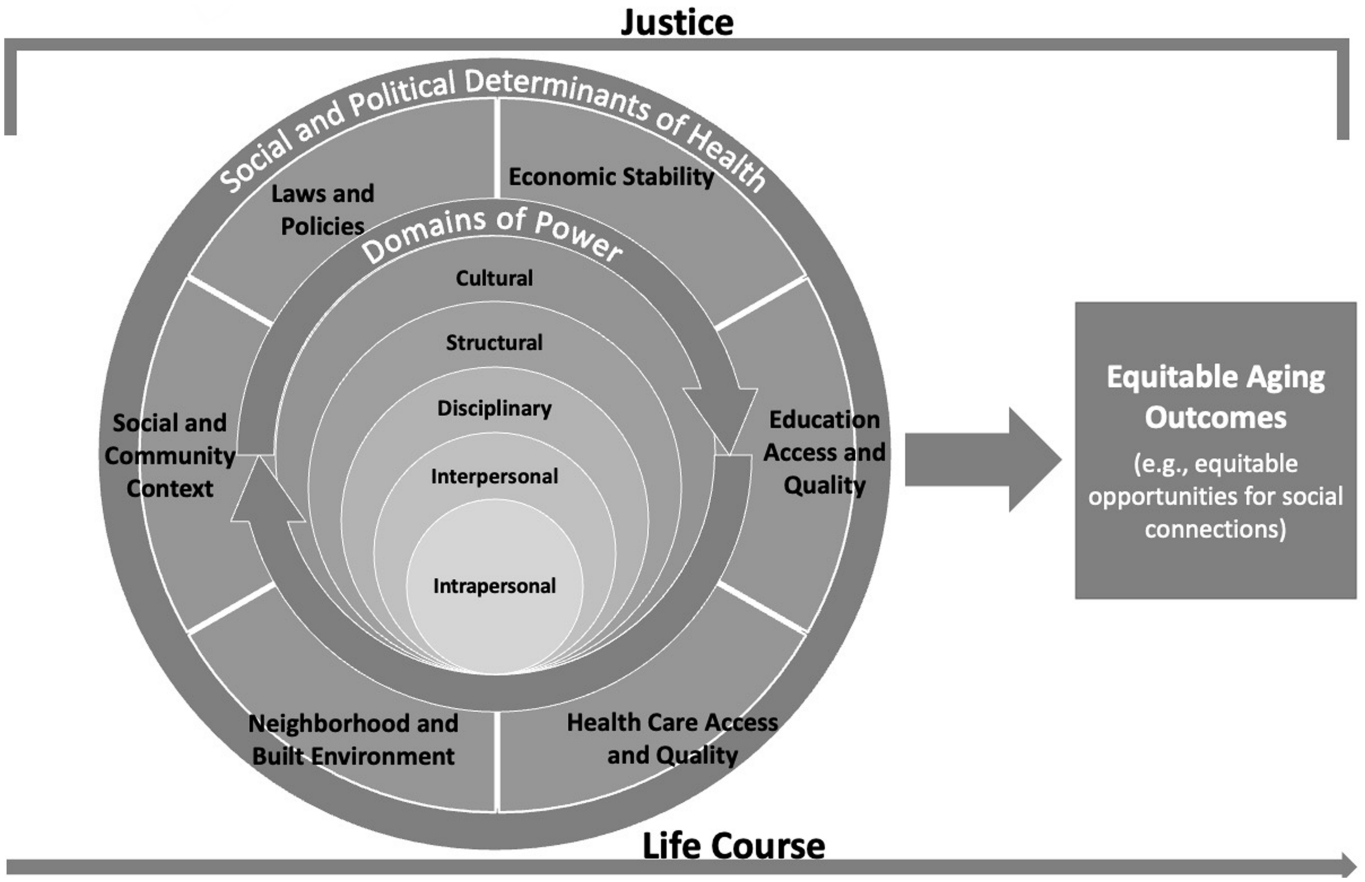
Equitable aging in health conceptual framework.

The Equitable Aging in Health Conceptual Framework builds on the contributions of prior scholarship in power and social and political determinants of health to present a new framework for considering how these concepts work together across the life course. This framework presents a tool to visually identify and map the ways that domains of power can shape social and political determinants of health and a blueprint for conceptualizing interventions that infuse justice. We argue that interventions that infuse justice must inherently address power, and this conceptual framework aims to help researchers, policymakers, and practitioners accomplish that objective. See Figure 1.

This conceptual framework foregrounds power as the engine that drives social and political determinants of health and potential interventions for equitable health outcomes across the life course. As described above, the five domains of power (structural, cultural, disciplinary, interpersonal, intrapersonal) play pivotal roles in social determinants of health—the structural and intermediate contexts in which people are born, grow, work, live, and age as well as the systems and structures that impact the conditions of their daily lives. They are overlapping and intersecting forces that can shape economic stability, education, physical environment (also referred to sometimes as the neighborhood and built environment), healthcare, and social and community context. For example, cultural norms about family (cultural domain of power) have been perpetuated in public policies and aging services (structural domain of power) about who is considered eligible for caregiver benefits that could reduce social isolation and loneliness among racially minoritized older adults who may rely more on intergenerational kinship relationships or families of choice. Disparities in benefits also serve to coerce racially minoritized older adults into caregiving arrangements that may increase social isolation or loneliness (e.g., paid care by strangers) because of eligibility requirements (disciplinary domain). Based on past experiences of discrimination, racially minoritized older adults may expect bias (intrapersonal domain) by strangers and be less receptive to or opt not to receive services that could minimize loneliness or social isolation (interpersonal domain). All five of these domains of power interact in complex ways to produce inequitable outcomes for racially minoritized older adults experiencing loneliness or social isolation, particularly in the social and community context (but also in health care access and quality) of the social determinants of health framework.

These five domains of power also shape political determinants of health and the public policies that can produce health inequities or health equity. Dawes (37) argues that public policies are the source of social determinants of health that produce health inequities. We do not dispute the significant role that public policies play in shaping social determinants of health. Yet, in this conceptual framework, we place public policies on the same level as social determinants of health to underscore the wide range of actions and activities outside of public policies that can help produce equitable outcomes. For example, if a local government is unwilling to subsidize rideshare or private taxis for individuals who no longer have public transportation options to access a grocery store, other community-based actions can emerge. Community coalitions may invoke community assets like well-connected churches, community gardens, and community volunteers to organize neighborhood farmer’s markets or food co-ops that could include food delivery, especially to community members with elevated health needs. Even with public policies in effect, community efforts can further strengthen their impact to help ameliorate health inequities.

To produce equitable outcomes, interventions must incorporate justice. Understanding the five domains of power can help researchers, policymakers, and practitioners envision possibilities for justice. Justice is defined in a variety of ways that incorporate principles of libertarianism (e.g., autonomy and self-governance), utilitarianism (e.g., greatest good for the most people), liberalism (e.g., fairness), capabilities approach (e.g., emphasis on quality of life), human rights (e.g., eliminating violence and intimidation), Black Feminist (e.g., liberation, dismantling oppression), Indigenous (e.g., healing, addressing colonialism), and Marxism (e.g., labor, production, eliminating class struggle), among other ideas. This article does not argue for any particular paradigm of justice but instead suggests that when considering various interventions, one should be mindful of what paradigm(s) of justice they are using and how this paradigm(s) can address power inequities that shape the social and political determinants of health for the targeted health issue. One intervention is not sufficient to address the multitude of power inequities and disparities that currently exist, and thus, this framework aims to serve as a tool to envision multiple interventions across the life course that can evolve and interact in ways that fuel equitable health outcomes as one ages.

When designing interventions through an Equitable Aging in Health Conceptual Framework, we recommend that researchers and public health and social service providers adopt a reflective approach that incorporates critical inquiry throughout the design process. This critical inquiry should involve questions that consider which paradigm(s) of justice an intervention will foreground and how it will incorporate the five domains of power to understand how these domains intersect and shape the goals of the intervention. For illustrative purposes, we pose several questions below that could be considered for health-related interventions using this framework:

1. ***Cultural Domain***: What strategies elevate values, meanings, interpretations, and ideologies in health-related interventions for a target population?
2. ***Structural Domain***: What policies and organizational changes are needed to facilitate equitable access to services?
3. ***Disciplinary Domain***: How can interventions address informal or subtle rules or practices in government or organizational bureaucracies that shape equity (or inequities) in health-related services.
4. ***Interpersonal Domain***: How can everyday interactions and relationships be restructured to reduce discrimination, bias, or other disparities?
5. ***Intrapersonal Domain***: What strategies can empower individuals, strengthen positive self-image, and address internalized stigma?

We also encourage questions that consider how targeted populations can be integrated into intervention design and/or feedback about an intervention designed by outside researchers, clinicians, and other policymakers and practitioners. Finally, we encourage researchers, practitioners, and policymakers to consider long-term sustainability and how that may require evolution over time (e.g., how should an intervention evolve as power dynamics change in various contexts and over time?).

The Equitable Aging in Health Conceptual Framework is meant to be a dynamic figure that can be adapted to fit the needs of particular health issues, populations, or contexts. Domains of power at the micro-level are nested in domains of power as they become more macro-oriented. However, the domains are interactive and interconnected. And, there may be times when one domain may dominate over another. For example, in a given circumstance, structural and cultural domains of power may overshadow other domains of power in the ways that they shape social and community context during a global pandemic (e.g., experiences of quarantine among Muslims in Nepal). In that situation, policymakers may want to consider these sections of the figure larger or at least devote more attention to these domains to identify the best interventions that produce equitable outcomes for older Muslims quarantining in Nepal.

The case examples below illustrate six different ways in which researchers, policymakers, and practitioners could use the Equitable Aging in Health Conceptual Framework to understand how power shapes certain social and political determinants of health and how a particular intervention that considers power can infuse justice to better achieve equitable outcomes for older adults. The first case example proposes the Rural–Urban Older Migrants’ Integration Initiative (RUOMII) to address social isolation among older rural–urban migrants in mainland China. The second case example presents the Cultural-Political Responsive Care Intervention to address social isolation and loneliness among Taiwanese Indigenous communities. The third case example presents the Personalized Assistance Plan Reform for Better Connectedness to address social isolation and loneliness among nursing home residents in Spain. The fourth case example poses cross-cultural legal clinics to address social isolation among non-European older migrants in Sweden. The fifth case example presents Ludotecas, a grassroots initiative to combat loneliness among older adults in Mexico. The sixth case example proposes an intergenerational arts-based program to build social connections and reduce loneliness among LGBTQIA+ older adults.

## Discussion

The case examples below are community-driven examples drawn from the personal and professional experiences of the authors, having lived and worked in each of these countries, including providing care, services, and policies for older adults in these regions. They also build on scholarly literature about challenges and promising programs relating to social isolation and/or loneliness among the region and populations they address. This article included two case examples from two different regions in Asia, Europe, and North America to provide both breadth and depth of application of this conceptual framework. Given that none of the authors have lived or work experience in other regions (e.g., Africa, South America), these global areas were not represented through case examples in this article. However, future applications of the Equitable Aging in Health Conceptual Framework in these regions could provide further insights into how aging inequities manifest in different cultural, political, and economic contexts. Researchers and practitioners in these regions are encouraged to adapt the framework as needed to address determinants of aging disparities and inform interventions for equitable aging outcomes.

### Case example 1: Social isolation among older rural–urban migrants in mainland China

Social isolation among community-dwelling older adults in urban areas of China has become a significant concern, particularly against the backdrop of the country’s demographic shift toward an aging society (65). In this context, the plight of a specific subset of the older population—rural–urban older migrants—has been somewhat neglected. During China’s rapid urbanization, these older migrants have moved to cities to live with their working children and assist them by taking care of their grandchildren and providing family support. As they age, their health and social needs escalate, yet their social networks and access to local health resources become increasingly inadequate in their new urban environments (66). Compared to their local counterparts, rural–urban migrants face heightened vulnerability as newcomers due to the unequal distribution of health and social welfare resources and discrimination, which is exacerbated by China’s household registration (*hukou*) system (67). This system contributes to their exclusion in urban settings, placing them at a disadvantage in accessing necessary services and integrating into the new environment (68). This case example presents an innovative perspective to analyze how interventions that foreground power could address disparities in social isolation among rural–urban older migrants in the hukou system.

#### Impact of social and political determinants of health on social isolation

The hukou system is a household registration framework that categorizes Chinese citizens based on their place of residence, differentiating between urban and rural (69). This system profoundly affects individuals’ access to social services and resource distribution, which can influence interventions to address social isolation. For example, after moving to urban areas, many rural–urban migrants do not obtain urban status. Their rural hukou status places them at a significant disadvantage compared to urban residents, leading to disparities in pension and health care benefits that exacerbate their economic instability (70). Limited access to social security and health care services further impedes their participation in community life and access to essential resources. Additionally, discrepancies in pension systems and a lack of retirement savings push many migrants toward poverty. Their situation is worsened by lower socioeconomic status, restricted social welfare access, educational and lifestyle differences with urban residents, and severe restrictions in health and welfare services due to their hukou status (66, 69).

Moreover, the social exclusion faced by rural migrants in urban environments limits their engagement in social activities, contributing to isolation and discrimination (67, 68). The lack of inclusion of older adults from rural backgrounds in urban design, coupled with alienation stemming from their transition to urban settings despite traditional support norms, calls for urgent attention. These challenges underscore the critical need for inclusive urban planning and support systems that address the complex barriers confronting rural–urban migrant older adults that can perpetuate social isolation.

#### Power inequities

Rural–urban migrant older adults in China face a multifaceted challenge of social isolation, shaped by diverse domains of power. Culturally, transitioning from rural to urban settings disrupts established family support mechanisms, and stigma associated with mental health and aging deters many from seeking assistance. Structurally, the hukou system places significant obstacles in their path, restricting access to vital services and intensifying feelings of dependence and isolation. Disciplinarily, there are stringent expectations for self-sufficiency and adherence to urban family caregiving norms, which leave those lacking immediate family support in a precarious state of isolation. Interpersonally, discrimination against rural migrants impairs their relationships with health care providers and community members, further limiting their access to necessary support. Internally, these individuals might absorb societal prejudices against rural migrants and older adults, leading to self-isolation and diminished self-esteem. To address the issue of social isolation among rural–urban migrant older adults effectively, a holistic approach is essential, promoting inclusive and culturally sensitive policies and interventions that facilitate their integration and enhance their well-being in urban settings.

#### Proposed intervention: rural–urban older migrants’ integration initiative

A study using national survey data in China has shown that improved community-level services and enhancements to the neighborhood and built environments can significantly reduce social disconnectedness and loneliness while improving life satisfaction among older adults (71). Based on these findings, the proposed intervention, the Rural–Urban Older Migrants’ Integration Initiative (RUOMII), is a targeted community social service program designed to address inequities in social isolation experienced by rural–urban older migrants in China.

At its core, RUOMII seeks to enhance the quality of life among these individuals by facilitating their integration into urban communities, ensuring access to essential services, and promoting social inclusion and engagement. The key components of this intervention are as follows. (1) *Community integration centers*: These centers will be established in urban areas with high populations of rural–urban migrants. These centers will serve as hubs for social, educational, and health-related activities tailored to the needs of rural–urban older migrants. Offerings will include digital literacy classes, health workshops, and cultural exchange events. (2) *Health and wellness services*: Through collaborations with local health care providers, the program will offer accessible health screenings, mental health support, and navigation assistance to help older migrants understand and navigate urban health care services and the urban health insurance system. (3) *Social networking and mentorship*: RUOMII will facilitate mentorship programs, pairing newer migrants with longer-term residents to share experiences, advice, and support. Social networking events will be organized to foster community connections and friendships.

**Figure 2:**
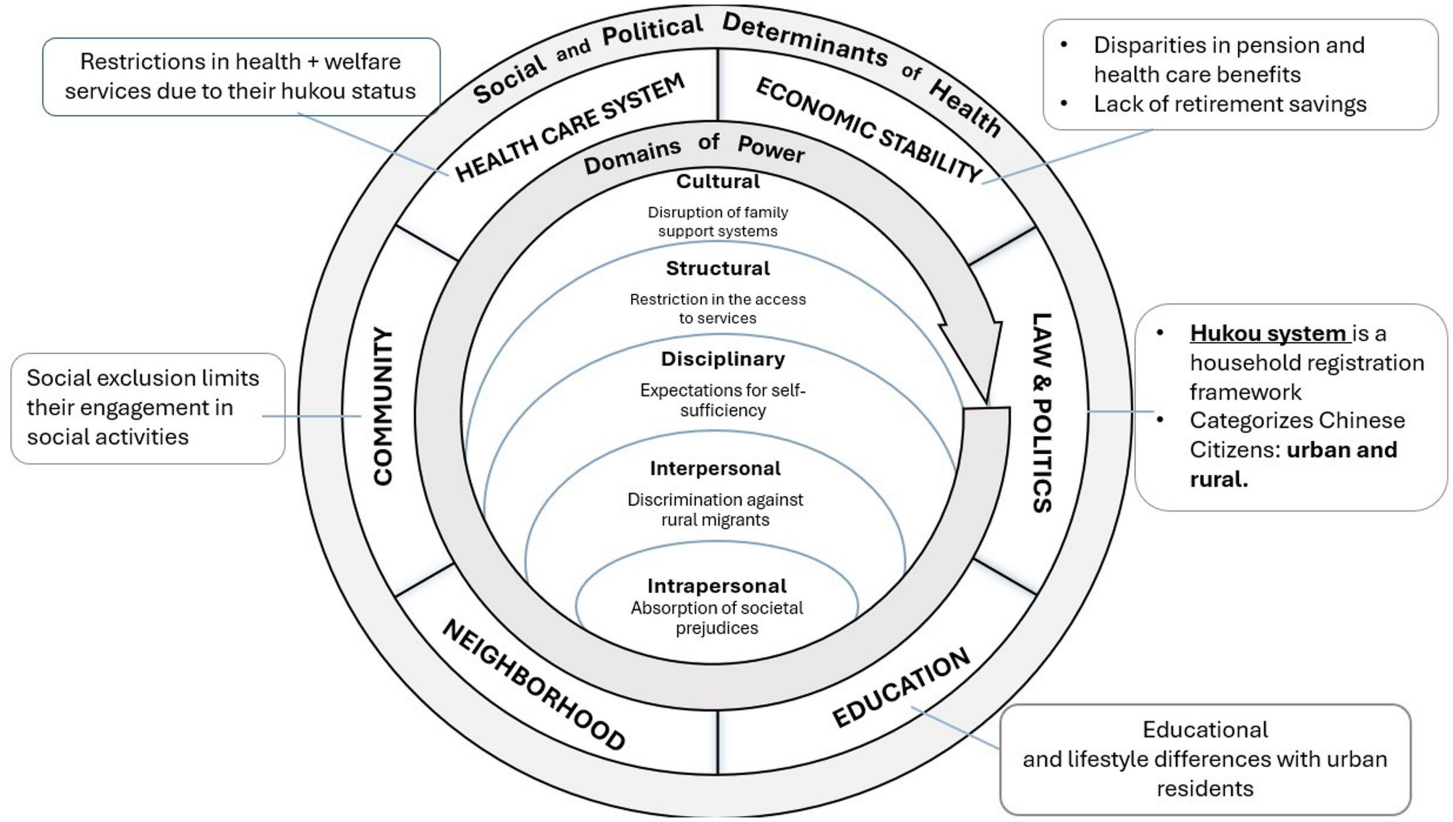
Rural–urban older migrants’ integration initiative (RUOMII) in urban mainland China.

This intervention aims to enhance the quality of life of individuals by focusing on community social service programs and improving the built environment. It will promote integration, ensure access to essential services, and encourage social inclusion and engagement. By incorporating social work principles of justice, this approach emphasizes equality, rights, advocacy, and the importance of participatory and collaborative methods (72). These methods not only affirm personal agency and diversity but also aim to address inequities in the following five domains of power, centering on diversity, equity, and inclusion. See Figure 2.

***Cultural Domain***: RUOMII will address cultural challenges faced by migrants through cultural exchange events and programs that honor both rural and urban traditions. By valuing the cultural backgrounds of older migrants, the initiative will help maintain their identities and support adaptation to an urban culture, thus mitigating cultural shock and stigma associated with mental health and aging. These programs will also help preserve important aspects of traditional family support structures in the urban context.

***Structural Domain***: The establishment of community integration centers and provision of health and wellness services will target the structural barriers imposed by the hukou system. By ensuring access to health screenings and mental health support regardless of hukou status, RUOMII will work around structural limitations, reducing dependence and isolation. This will directly address inequities in accessing essential urban services and health care.

***Disciplinary Domain***: The program’s social networking and mentorship components will challenge the disciplinary expectations of self-reliance and urban family care standards. By creating a support network that includes mentorship from longer-term residents, RUOMII will provide a sense of belonging, community support, and opportunities for older adults to make new friends. This intervention will reduce vulnerability to isolation among older migrants by fostering a collective approach to care and support.

***Interpersonal Domain***: RUOMII’s social networking events and mentorship programs also will address interpersonal power dynamics, particularly discrimination against rural migrants. By fostering connections between newer migrants and longer-term residents and organizing community events, the initiative will promote understanding and reduce biases. Improved relationships with health care providers and community members could enhance older migrants’ access to resources and support, thereby reducing social isolation.

***Intrapersonal Domain***: RUOMII’s offerings, such as digital literacy classes and health workshops, will empower older migrants by boosting their self-efficacy and knowledge and tackling internalized biases and low self-esteem. By providing skills and information, RUOMII can help individuals feel more capable and confident in navigating urban life, counteracting the effects of societal biases against rural migrants and aging.

In summary, RUOMII’s integrative approach to social, recognition, and distributive justice will address the complex nature of social isolation among rural–urban older migrants. By acknowledging and addressing the various forms of inequity and exclusion these migrants face, RUOMII aims to create a more inclusive and just society. This comprehensive approach aims to mitigate immediate impacts of social isolation and underlying structural and societal factors contributing to this issue to foster a community where everyone, regardless of their background or migration status, can thrive.

### Case example 2: Social isolation and loneliness in Taiwanese indigenous communities

#### Power inequities: colonialism and aging

Indigenous Taiwanese people experience multi-level hindrances in society based on the historical oppressions prompted by prolonged colonialism. Ever since encountering the Dutch voyage fleets in the 17th century, Indigenous Taiwanese have been ruled by several colonial policies of plunder, exploitation, and impoverishment, resulting in massive loss of lives, land, resources, culture, knowledge, and identity. These adverse historical legacies have exacerbated health disparities for Indigenous Taiwanese people across the life course and domains of power, including through experiences of social isolation and loneliness in aging.

In the cultural domain, Indigenous Taiwanese epistemologies and ontology have been gradually eroded by coercive assimilation, including prohibitions of using native languages, traditional religious ceremonies, and hunting activities. Losing and devaluing cultural practices have prevented Indigenous elders from connecting with younger generations and perpetuated social discrimination and stigma toward Indigenous people (73). Structurally, industrial urbanization accompanied with modernization has propelled the migration of Indigenous youth from rural to urban areas, causing isolated Indigenous elders who stayed in the villages to have fewer intrafamilial caregivers (74). Low socioeconomic status rendered by the appropriation of natural resources and exploitation of labor has further impacted Indigenous people’s access to adequate, quality health care and restricted engagement in social activities and networks. In the disciplinary domain, the Taiwanese government has implemented elder care programs that omit Indigenous epistemology and axiology and coerce Indigenous elders to adopt foreign ideologies of caregiving to participate (75). Public services based on majority hegemony are not only ineffective in achieving community-based care in Indigenous communities but have become new apparatuses that discipline their behavior as a new form of welfare colonization (76, 77). Interpersonal ageism toward Indigenous elders occurs when care providers define them as “frail” patients without contextualizing health conditions within Indigenous community assets. Elders in many Indigenous societies play pivotal roles as superior knowledge keepers and influential members who carry sacred knowledge (78), including knowledge about building and maintaining robust care support networks. Ignoring their social identity hinders potential social connections. Intrapersonally, constant invalidation toward Indigenous people’s existence can cause identity issues, self-doubt, and hypervigilance (79) that exacerbates the risk of isolation and loneliness in aging.

#### Proposed intervention: cultural-political responsive care

**Figure 3:**
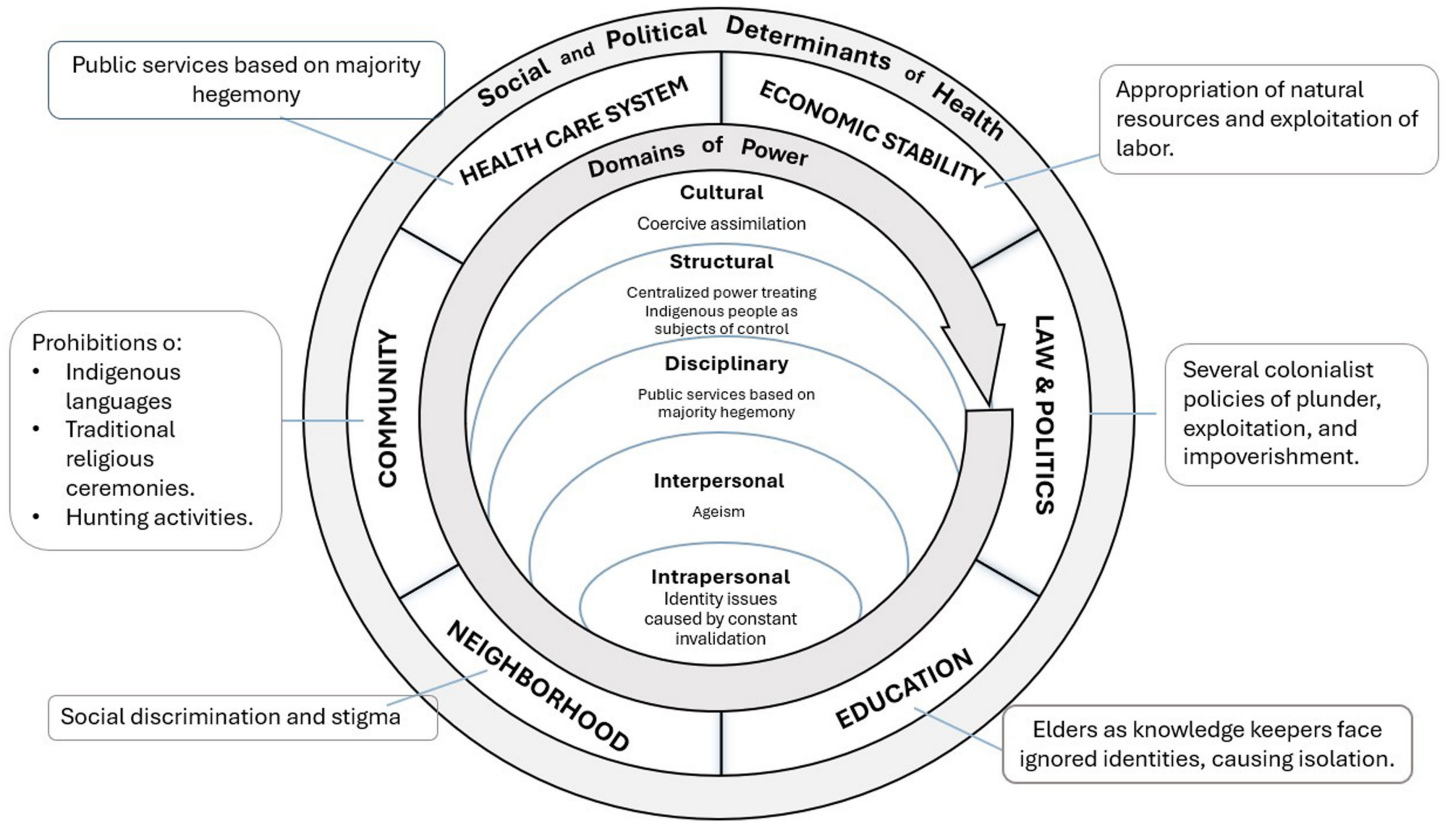
Cultural-political responsive care for indigenous Taiwanese elders.

Despite a long history of oppression, preexisting cultural knowledge could be leveraged to form robust social networks that prevent social isolation and loneliness. Derived from the equitable aging framework, this case example proposes Cultural-Political Responsive Care as an intervention for care decolonization. This intervention merges traditional cultural knowledge, collectivism, and self-determination to address inequities in the five domains of power and provide an intervention centered on decolonization in community-based care for Indigenous elders. See Figure 3.

Cultural-Political Responsive Care aims to establish culturally adequate care by acknowledging and responding to structural power inequities, based on understanding the cultural and political context embedded in care practice, especially when working with historically marginalized people, including but not limited to Indigenous elders, Black older adults, immigrants, and LGBTQIA+ communities. Cultural-Political Responsive Care combines three imperative components: cultural humility, cultural safety, and political devolvement. (1) *Cultural Humility*, inspired by Madeleine Leininger’s Transcultural Nursing Theory (138), involves knowing and understanding different cultures in health-illness caring practices, beliefs, and values to provide meaningful and efficacious nursing care services in people’s cultural health-illness context (141). Humility training includes building cultural awareness, generating cultural knowledge, applying cultural skills, and engaging culturally diverse others in practice settings and contexts (80). Cultural humility requires immersion and cultivating the epistemology and cosmology of Indigenous people to form a cultural consciousness of social relationships and local networks in practice (66). (2) *Cultural Safety* means acknowledging power inequities between providers and care recipients that stem from a history of colonization and addressing related biases, attitudes, assumptions, stereotypes, prejudices, and structures that may affect the quality of care. The scope of providers is not limited to individuals but includes institutions such as government departments, hospitals, clinics, and schools (81). Cultural safety requires healthcare professionals and their associated healthcare organizations to reduce bias that is embedded in healthcare practices, achieve equity within the workforce and working environment (82), and empower care recipients through health information transparency (75). (3) *Political Devolvement* is necessary to respond to the uniqueness of highly regulated disciplinary power in Taiwan’s care system. Elder care policies and regulations in Taiwan have developed under the long history of service professionalization and management accountability in the contracting model of social welfare that excludes Indigenous political participation. Thus, the political sustainability of Indigenous people’s self-determination aims to support the decentralization of the public-funded care system and encourage the community’s participation in program design and provision to create localized and diverse care models and methods.

Cultural-Political Responsive Care infuses Indigenous theories of justice that center decolonization to respond to inequities in the five domains of power through these three aspects (cultural humility, cultural safety, and political devolvement), as described below.

***Cultural Domain***: By foregrounding Indigenous knowledge, Cultural-Political Responsive Care addresses power inequities in the cultural domain by elevating Indigenous elder networks and connectedness practices. Reestablishing the practices stemming from cultural values and knowledge is the foundation of decolonization in social and health-related services for Indigenous people.

***Structural Domain***: Political devolvement of care policy liberates the centralized power that treats Indigenous people as the subject of control and regulation. It further secures Indigenous people’s participation in the legal decision-making process and legitimizes Indigenous knowledge of care at the structural level.

***Disciplinary Domain***: Cultural safety creates a partner relationship for institutions and elders to collaborate on designing service programs and provisions with mutual respectfulness and collectivism to reduce power inequities between government and Indigenous people on the cultural and disciplinary levels. Service programs are thus designed in culturally responsive and inclusive ways that move away from regulations of control and surveillance (e.g., who is “worthy” of particular services).

***Interpersonal Domain***: Cultural Humility equips practitioners with awareness of cultural differences that center Indigenous knowledge and value and reconstructing the partnership relation between practitioners and recipients, which reduce discrimination and stigma based on Western hegemony and ignorance at the interpersonal level.

***Intrapersonal Domain***: Awareness of cultural differences through recognition of Indigenous knowledge and uniqueness benefits practitioners in considering the perception of Indigenous recipients. Practicing cultural care reduces psychological stress at the intrapersonal level.

Ultimately, the Cultural-Political Responsive Care Intervention illustrates how infusing justice to address power inequities can strengthen care programs for Indigenous elders in Taiwan.

### Case example 3: Social isolation and loneliness in Spanish nursing homes

In Spanish nursing homes, the problem of social isolation and loneliness among residents is pervasive (83). Studies consistently reveal alarmingly high levels of social disconnection, which exceed those observed in community-dwelling older adults (84, 85). Loneliness is also experienced differently depending on the individual circumstances, or positionalities, of residents (86). This underscores both the impact of social determinants of health on residents’ isolation and the need to address underlying power imbalances in these settings. Indeed, despite efforts by policymakers to foster social connections, ignoring these dynamics undermines the effectiveness of such initiatives. Against this background and to exemplify another equitable intervention that infuses justice across different domains of power, this third case example involves a normative reform that prioritizes contextualized justice and personalized support to rectify power inequities in Spanish nursing homes.

#### Impact of social and political determinants of health on social isolation

Social isolation in nursing homes is multifaceted. Social determinants of health such as economic stability, education, cognitive and motor abilities, built environment, family support, and community involvement create disparities among residents, affecting their social connectedness. For example, economic stability influences the choice of care homes, as families must decide between facilities with more staff and a manageable workload versus other centers with poorer conditions. This disparity in workload impacts the time caregivers allocate to interacting with residents, and research underscores that these interactions are important for fostering a sense of connectedness (87). Similarly, educational disparities can limit residents’ ability to negotiate social opportunities, such as extended visiting hours. These negotiations are essential given that there are no minimum legal standards governing visitation or outings in the Spanish residential context. In addition, the surrounding environment of nursing homes influences access to social activities and community participation (88); for example, proximity to parks and community centers may promote social participation (89, 90), while transportation limitations may hinder connection to the broader community (91).

#### Power inequities

Analysis of the social determinants of isolation in nursing homes in Spain reveals intersections with power dynamics that influence residents’ experiences. Intrapersonal power dynamics, influenced by past experiences and generational norms, affect residents’ perceptions of their rights and abilities to advocate for their needs, which may deter them from seeking social interactions that alleviate loneliness (92). Interpersonal power dynamics between residents, family members and caregivers also influence social connections within nursing homes. Factors such as residents’ previous roles in the community, gender (93), staffing ratios, room layout, and rules about the use of common areas shape this domain of power. For example, in many Spanish nursing homes, rules require residents to retire to their individual rooms after dinner, limiting any social interaction after 8 pm.

Structural power is reflected in organizational policies, such as strict visiting rules that coincide with families’ work hours, limiting residents’ ability to connect and increasing their isolation. Cultural power, intertwined with structural power, stigmatizes nursing home use due to traditional social norms and constructs about aging, gender, and caregiving, reinforcing the cultural preference for family care (94). This perpetuates the perception that nursing home residents are less valued or deserving of community engagement. Recognizing and addressing these complex power dynamics can promote more equitable access to services and social support networks in nursing homes, thereby reducing social isolation, and improving overall well-being.

#### Proposed intervention: personalized assistance plan reform for better connectedness

Research indicates that loneliness and isolation are highly personal experiences influenced by individual and structural circumstances (86). However, various intervention programs have demonstrated significant success in alleviating these issues (95). Building on these findings, the following intervention infuses principles of critical contextualist justice to foster social connections within care homes: the Personalized Assistance Plan Reform for Better Connectedness.

The Personalized Assistance Plan (PAP) is a mandatory tool established by the Social Services Laws of all the Autonomous Communities of Spain, which guarantees it as a right for all residents. Developed in collaboration among professionals, residents, and family members, the PAP consists of personalized care strategies and health interventions for each resident. It focuses on health outcomes, mobility, and cognitive abilities, which involves the inclusion of dietary routines, physical therapies and exercise, among other aspects. However, it often lacks emphasis on interventions targeting social isolation. Our normative reform proposal aims to improve the PAP’s approach to social connection mechanisms.

This policy intervention includes the following key components: (1) *Mandatory Assessment:* Mandatory assessment of social isolation within the PAP to identify residents’ needs and preferences in this sphere; (2) *Individual and Group Activities:* Tailored group and individual activities alongside health routines within the PAP (e.g., video calls with families and volunteers, board game championships, reading groups); (3) *Collaboration:* Leveraging the collaborative nature of the PAP by involving experts, residents, and families in planning social activities to promote active resident participation, which has been found to reduce loneliness (86); and (4) *Customization:* Enabling the Personal Assistance Program (PAP) to customize facility regulations. This reform encourages adapting rules such as visiting hours or access to common areas after dinner according to residents’ capabilities and preferences. This approach harmonizes and balances safety and the duty of care, traditionally central to nursing homes, with social interaction and residents’ rights, empowering them to negotiate their level of independence.

**Figure 4:**
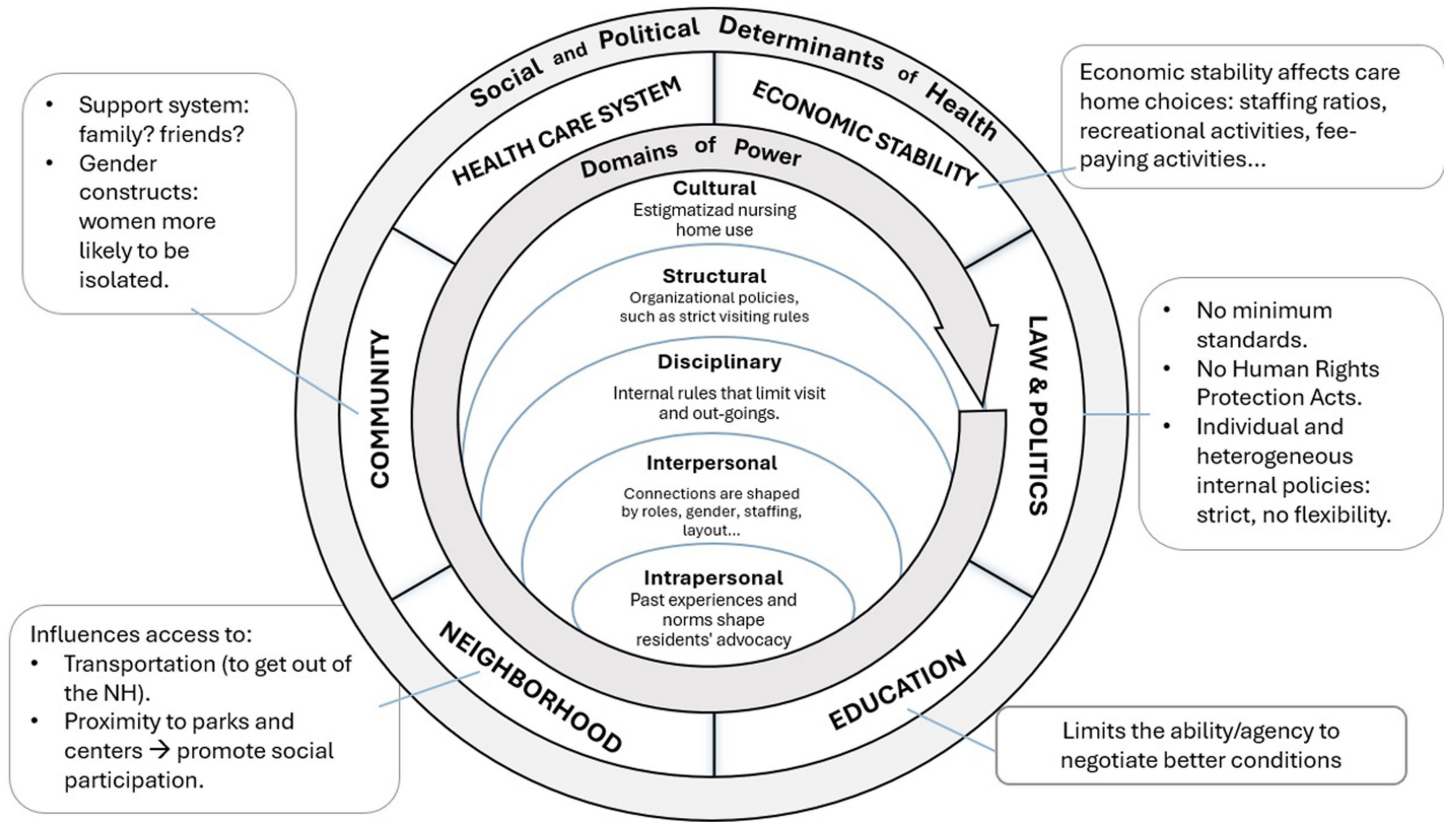
Personalized assistance plan reform for better connectedness in Spain.

The proposal aligns with Critical Contextualism, a justice paradigm emphasizing context-specific analysis for tailored interventions and fair outcomes (96, 97). This approach recognizes that justice is contingent upon the specific circumstances, allowing for nuanced responses to individual needs. By focusing on the unique contexts of each resident, the intervention aims to challenge past experiences of disempowerment and promote agency and belonging. In doing so, it seeks to address power imbalances and foster equitable health outcomes for all residents, in the following ways. See Figure 4.

***Cultural Domain***: Collaboratively developed care and social strategies within the PAP respects residents’ cultural backgrounds and preferences, acknowledging the importance of cultural identity in promoting a sense of belonging. By embracing diversity and inclusivity, the intervention aims to mitigate cultural biases and cultivate a more responsive care environment. Furthermore, involving families in the social planning process enhances family engagement and support networks, alleviating feelings of shame and stigma among families and reducing resident isolation.

***Structural Domain***: This reform prioritizes social interaction and implements a case-by-case system to address power imbalances inherent in existing practices, such as overly strict visiting hours. By fostering flexibility and autonomy, the reform aims to create a more inclusive and supportive environment for residents.

***Disciplinary Domain***: The reform takes advantage of the transparent mechanisms already in place under the periodic PAP reviews (required under the social service laws) to gather residents’ opinions and feedback in a less hierarchical and more collaborative manner. By prioritizing residents’ voices and experiences, the reform empowers residents to actively participate in decision-making processes and advocate for their needs as opposed to penalizing residents through informal systems of surveillance.

***Interpersonal Domain***: Through tailored group and individual activities, the intervention seeks to foster social connections and community engagement among residents. Increased family involvement and flexible scheduling further support residents’ social well-being, enhancing their overall quality of life.

***Intrapersonal Domain***: The PAP’s emphasis on individualized care and resident participation empowers residents to take an active role in their own care, social planning, and decision-making processes. By recognizing residents’ autonomy and agency, the intervention promotes self-determination and personal growth, enhancing residents’ sense of control, dignity, and self-worth.

In summary, the Personalized Assistance Plan Reform for Better Connectedness illustrates how infusing principles of justice to address power inequities into an intervention can create a more inclusive and equitable environment in nursing homes. By addressing diverse challenges and disparities, it seeks to alleviate immediate social isolation effects while tackling underlying structural factors. Through personalized care strategies and enhanced social connections, the intervention aims to foster a sense of belonging and well-being for every resident.

### Case example 4: Social isolation among older non-European migrants in Sweden

Social isolation among older migrants in Sweden is a growing concern, especially with the country’s aging population and increasing migration trends (98). While much focus is placed on urban migrant populations, the challenges faced by older non-European migrants are often overlooked. These individuals, who typically migrate to join family or escape conflict, experience deteriorating social connections due to cultural differences, language barriers, and systemic inequalities within Sweden’s welfare system, which often fails to meet their needs (99). The case example explores this reality, applying our framework to an intervention that addresses power dynamics and reduces disparities.

#### Impact of social and political determinants of health on social isolation

Older migrants in Sweden, particularly those from non-European backgrounds, face heightened risks of social isolation due to social and political determinants of health, including economic instability, language barriers, and inadequate access to culturally tailored healthcare (99, 100). Unlike their Swedish-born counterparts, many older migrants have no or insufficient pensions, limiting their ability to afford social activities and fully participate in community life (98). This economic disadvantage increases feelings of alienation, as older migrants may feel disconnected from society, unable to integrate due to their financial challenges (101). At the structural level, Sweden’s welfare system, often designed for a homogeneous population, fails to address the unique needs of older migrants, especially those who arrive later in life. These individuals experience “double isolation” due to language and cultural barriers, which exacerbates their marginalization and limits access to essential services (102). Furthermore, the political framing of older migrants as a “social risk” discourages the development of inclusive policies and advocacy (99).

#### Power inequities

Power influences material conditions, social dynamics (57), and knowledge production (58), upholding societal hierarchies. For older non-European migrants in Sweden, social isolation is shaped by five intersecting power dynamics, which affect their access to resources, influence policy, and limit social participation. Cultural power dynamics emerge through societal stereotypes that shape how migrants are perceived, often leading to misunderstandings and discrimination. Furthermore, a lack of cultural competence in healthcare and caregiving further isolates older migrants, as they may face communication barriers and care that does not respect their cultural needs. This reinforces feelings of alienation and exclusion. Structural power further isolates migrants, as Sweden’s welfare system, designed for a homogeneous population, overlooks their unique needs, particularly around language and navigating complex administrative processes (103). Disciplinary power operates within healthcare systems, where the absence of culturally sensitive care discourages migrants from seeking necessary services, deepening their isolation. Intrapersonal power emerges as migrants internalize societal stigmas, hindering their ability to connect with others. Lastly, interpersonal power is disrupted due to the weakening of family support systems, a common consequence of migration and language barriers. These power dynamics reinforce exclusion, underscoring the necessity of a comprehensive approach to reduce inequities and promote social inclusion for older migrants.

#### Proposed intervention: cross-cultural legal clinics for older migrants

To address the legal barriers faced by older non-European migrants in Sweden, this case example proposes the establishment of Cross-Cultural Legal Clinics, which would provide free, culturally sensitive legal support. These clinics, operated by university law students under professional supervision, would focus on areas such as pensions, healthcare administrative processes, inheritance, and family law. Leveraging the diverse student body, many of whom are second- or third-generation migrants with a deep understanding of both Swedish society and the challenges faced by migrant communities, would ensure the services are relevant and culturally appropriate. Multilingual interpreters and cultural mentors would further enhance accessibility, creating a more inclusive and effective legal intervention for older migrants. This intervention not only addresses the legal barriers faced by older migrants but also plays an important role in reducing their social isolation by fostering trust, strengthening community ties, and empowering them to actively participate in Swedish society. Furthermore, these clinics could be complemented by community-based initiatives, such as digital literacy programs, to tackle the broader social and structural challenges that older migrants face.

**Figure 5:**
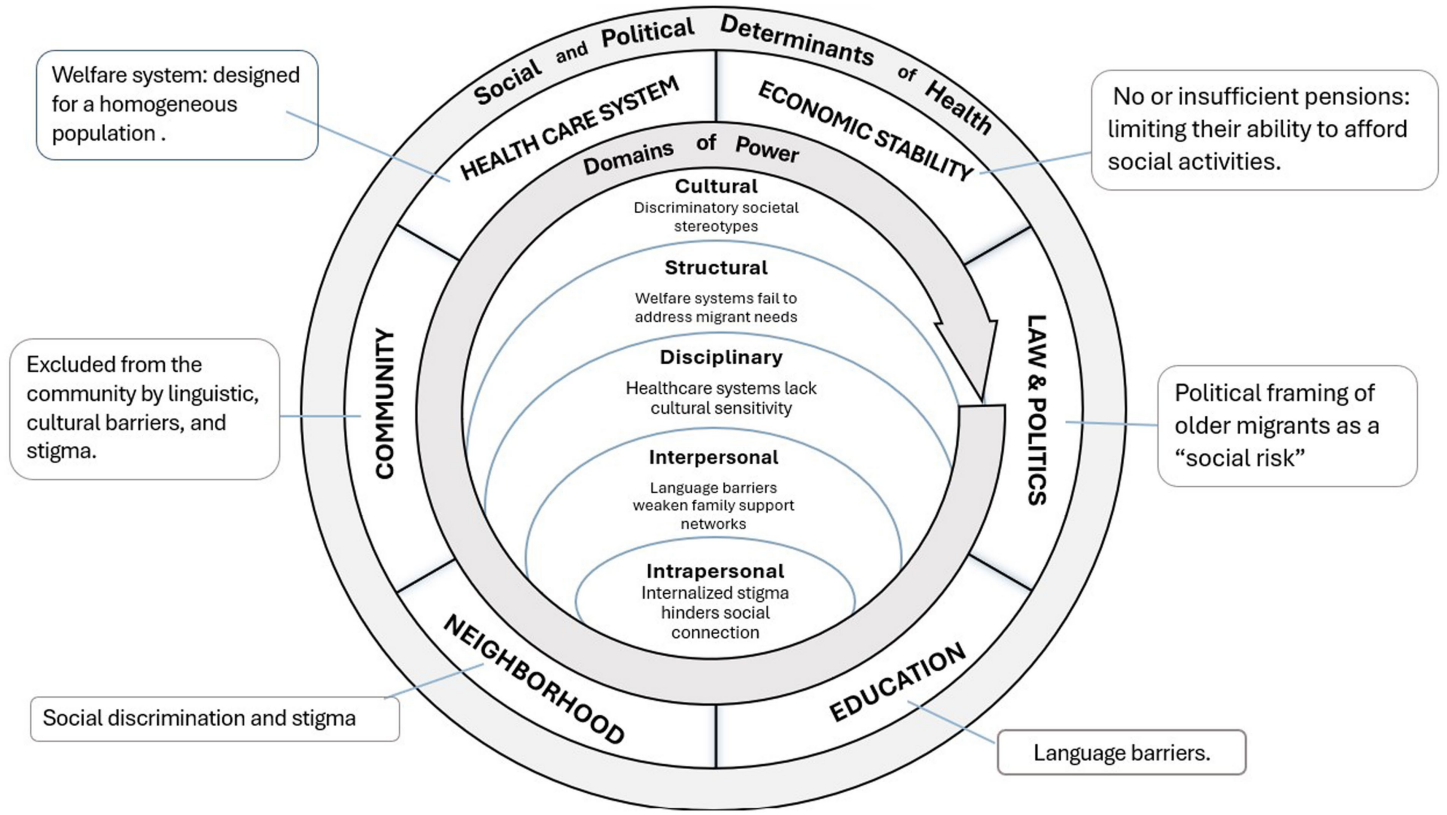
Cross-cultural legal clinics for older migrants in Sweden.

The Cross-Cultural Legal Clinics are grounded in Capabilities Theories of Justice, aiming to empower older migrants by providing the tools and support necessary to navigate the legal system and exercise their rights, thus promoting social inclusion and reducing isolation (104, 105). See Figure 5.

This intervention infuses justice in the domains of power in the following ways:

***Cultural Domain***: The intervention emphasizes cultural sensitivity by integrating interpreters and cultural mentors, alongside law students from diverse backgrounds who may be more familiar with the challenges migrants face. This ensures that legal advice is culturally respectful, fostering inclusivity and reducing the risk of alienation.

***Structural Domain***: By offering free, tailored legal services, the clinics challenge the rigid and often inaccessible legal systems. This flexible approach allows older migrants to engage with legal processes, overcoming language and cultural barriers.

***Disciplinary Domain***: The collaboration between law students, legal professionals, and migrants helps break down traditional power dynamics in legal services. This mutual learning environment promotes respect and ensures legal services are empowering for older migrants.

***Interpersonal Domain***: Building trust between legal service providers and older migrants helps strengthen community ties, reducing isolation and encouraging migrants to seek support, knowing their needs will be understood and addressed.

***Intrapersonal Domain***: The clinics equip older migrants with legal knowledge and resources, boosting their confidence and autonomy. This empowers them to navigate Swedish society more effectively and assert their rights, reducing feelings of marginalization.

The Cross-Cultural Legal Clinics present a comprehensive, rights-based approach to the legal challenges faced by older non-European migrants in Sweden. By addressing power imbalances across multiple domains, the intervention promotes social inclusion, equality, and greater participation in society.

### Case example 5: Addressing loneliness (“Soledad”) among older adults in Mexico through Ludotecas, grassroots, and intergenerational learning

Loneliness (“soledad”) and social isolation among older adults in Mexico have emerged as significant public health concerns due to shifting demographics, economic migration, and changes in family structures. Mexico’s older adult population (60+) currently comprises 12.3% of the total population, a figure expected to double to 22.5% by 2050 (106). Traditionally, familial caregiving has provided social support, but urban migration has disrupted multigenerational living, leaving many older adults socially disconnected. According to the National Survey on Health and Aging in Mexico, 35.4% of older adults reported experiencing loneliness, rising to 39.8% during COVID-19 (106). Research from Nuevo León found that 5% of older adults were at risk of or experiencing loneliness, with strong correlations between loneliness and sleep deprivation (107). These findings underscore the detrimental effects of loneliness and social isolation on mental and physical health, including increased risks of depression, cognitive decline, and cardiovascular disease (108).

While Mexico has government programs such as INAPAM cultural centers and day residences, access remains highly uneven, with rural and low-income older adults facing systemic barriers to participation. Older Mexican adults face both high poverty and high labor force participation rates, among the highest in the OECD, due to the country’s large informal economy. This economic vulnerability often limits their ability to access INAPAM’s services, which are more readily available in urban areas (109, 110). Additionally, older adults from Indigenous or rural backgrounds often face cultural stigma, limiting their willingness to engage in urban social programs (111). Digital exclusion further compounds these disparities, as many older adults lack access to digital technologies, hindering virtual socialization and necessary telehealth services (112).

Given these systemic inequities, there is an urgent need for culturally appropriate interventions that foster intergenerational engagement, socialization, and lifelong learning. The Ludotecas y Aprendizaje (Play & Learning) model provides a structured solution to address social isolation, cultural exclusion, and digital literacy barriers among older adults in Mexico.

#### Impact of social and political determinants of health on loneliness

Economic migration in Mexico has led to family separations, with younger generations moving to urban areas or abroad for work, leaving many older adults in rural communities. This migration disrupts traditional caregiving structures and reduces intergenerational interactions, increasing loneliness among older adults. For those who relocate to urban centers with their children, challenges in forming new social connections can persist, especially when cultural and linguistic differences create barriers to integration (109). Limited access to social and health services further exacerbates loneliness. While programs like INAPAM cultural centers offer engagement opportunities, financial constraints, geographic isolation, and transportation issues often hinder participation for many older adults. In rural areas, the absence of community spaces makes it difficult to establish new relationships. Indigenous older adults may also face cultural stigma or language barriers when accessing urban services, leading to further social withdrawal (111).

Digital exclusion compounds these challenges. Many older adults have limited formal education and lack the skills needed to use digital communication tools. As social interactions, healthcare, and government services increasingly move online, those without digital literacy are left disconnected from essential resources and support networks. While technology can potentially alleviate loneliness, without access or training, older adults remain at risk of chronic isolation (112). Addressing these disparities through culturally and contextually relevant interventions is essential to reduce loneliness and promote meaningful social participation among older adults in Mexico.

#### Power inequities contributing to loneliness in Mexico

Loneliness among older adults in Mexico is shaped by intersecting domains of power that limit access to social participation, resources, and well-being. Culturally, the decline of traditional multigenerational family caregiving models has led to weakened social networks, especially during COVID-19, while older adults from Indigenous or rural backgrounds face the stigma that limits their integration into urban social spaces. Structurally, the uneven distribution of social programs has created barriers to participation in socialization initiatives that increases risks of loneliness. Many INAPAM programs are concentrated in wealthier urban areas, leaving rural older adults without access to these essential services. Disciplinarily, aging is often framed as a period of dependence rather than active participation, discouraging older adults from engaging in educational, social, or digital inclusion programs. Societal expectations reinforce the belief that older adults cannot learn new skills, limiting their engagement in technology and community initiatives. Interpersonal power dynamics also create barriers to intergenerational connection, as technological and cultural gaps hinder communication between younger and older family members. Many older adults struggle to maintain relationships with children and grandchildren who have migrated, often due to technological divides or generational misunderstandings. Intrapersonally, older adults may internalize societal messages that reinforce helplessness and exclusion, leading to decreased self-esteem and voluntary social withdrawal. Many believe they are “too old” to participate in educational programs or social activities, limiting their community engagement. Addressing these intersecting power inequities requires an approach that empowers older adults by promoting social participation, lifelong learning, and intergenerational connection.

#### Proposed intervention: Ludotecas model

The Ludotecas model is a grassroots initiative designed to combat loneliness among older adults by integrating structured play, technology training, and intergenerational learning into community-based spaces. Rooted in local traditions and collective work, this intervention expands on existing ludoteca initiatives, particularly in Indigenous and underserved communities, to create inclusive environments where older adults actively participate, learn new skills, and foster meaningful social connections. Rather than positioning older adults as passive recipients of services, this approach emphasizes their role as knowledge-holders, mentors, and contributors to community life.

**Figure 6:**
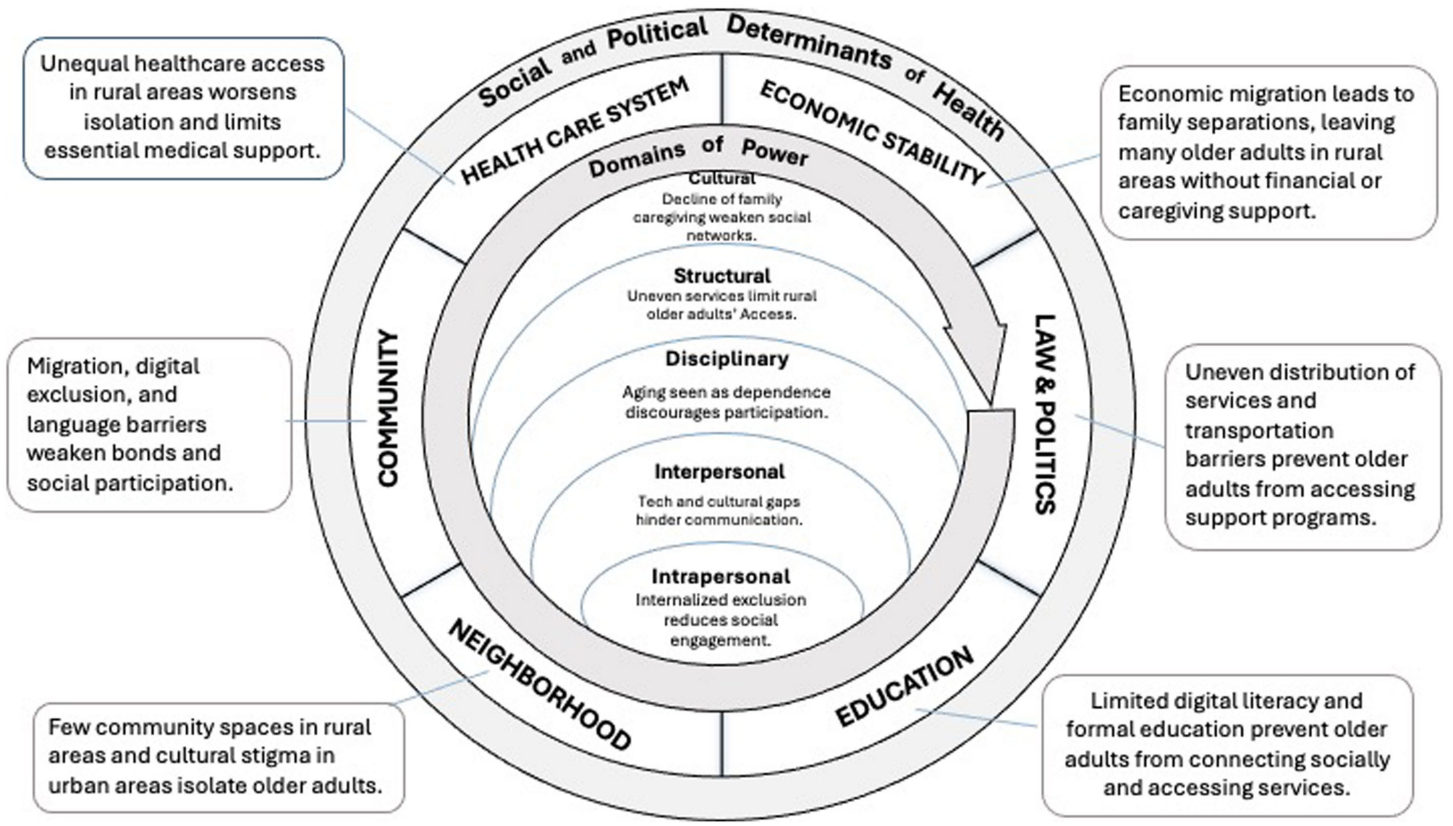
Ludotecas grassroots initiative for older adults in Mexico.

Building on successful community-based initiatives such as the Biblioteca y Ludoteca Comunitaria Ambulante de Comachuén and the Ludoteca y Aula de Medios en la Comunidad Mazahua, this intervention builds on intersectional theories of social justice to leverage existing culturally embedded education models to provide a structured response to loneliness among older adults (134, 135). Traditionally centered on children, this initiative already informally includes adults, mainly parents, and sometimes grandparents, who observe or participate on the sidelines. Expanding their role would transform ludotecas into intergenerational learning spaces, where older adults lead and actively participate in community activities. See Figure 6.

***Cultural Domain***: The Ludotecas model will be rooted in cultural traditions and reinforce cultural identity and knowledge-sharing. Through storytelling sessions, oral history projects, and traditional skill workshops, older adults will be empowered in their roles and strengthen social networks across generations. By maintaining mobile ludotecas and expanding them into permanent community spaces in Indigenous and rural communities, ludotecas will challenge urban biases that marginalize older adults and create affirming spaces that foster belonging.

***Structural Domain***: The program will expand access to social participation by providing community-driven spaces for structured play/experiential workshops, education, and lifelong learning, through local learning centers, universities, and library partnerships. By removing geographic and economic barriers, this initiative will broaden access to critical social and educational resources.

***Disciplinary Domain***: Ludotecas will actively challenge ageist narratives that portray older adults as dependent and incapable of learning, the program will redefine aging as a period of active participation. The inclusion of digital literacy training further reinforces this shift, equipping older adults with technological skills to connect with family members, access services, and participate in broader social networks, counteracting exclusionary norms.

***Interpersonal Domain***: The program will facilitate intergenerational dialog and connection, reducing technological and cultural gaps between younger and older generations. Through structured mentorship programs, digital learning exchanges, and community festivals, ludotecas will bridge generational divides and reduce stigma associated with aging. Ludotecas will offer free digital literacy workshops, teaching older adults how to use smartphones, social media, and video calls to stay connected with family members who have migrated.

***Intrapersonal Domain***: Ludotecas will empower older adults by providing structured learning environments that build confidence and self-efficacy. By participating in skill-sharing workshops, digital literacy classes, and volunteer initiatives, older adults can counteract internalized ageism and recognize their continued value within the community.

### Case example 6: Social isolation and loneliness among LGBTQIA+ older adults in the United States

LGBTQIA+ older adults in the United States have heightened risks for social isolation and loneliness related to historical exclusion from family formation (e.g., marriage, adoption, nonacceptance), discrimination, and social bias [e.g., (113–116)] (142). In response to these barriers, LGBTQIA+ communities have a long history of developing robust caregiving and support networks among families of choice (116, 117) and often across generations or age cohorts [e.g., (115, 118–120)]. This case example presents an intergenerational intervention that foregrounds justice and power and draws on this rich history of support.

#### Impact of social and political determinants of health on social isolation and loneliness

LGBTQIA+ older adults in the United States experience social, health, and economic disparities (121, 122, 123, 143) that contribute to higher risks of social isolation and loneliness for LGBTQIA+ older adults. Exclusion, bullying, and othering in educational spaces can also perpetuate feelings of loneliness and disconnection. Access to inclusive and affordable housing (as well as housing discrimination) have presented challenges for building easily accessible communities among families of choice for many LGBTQIA+ older adults [e.g., (123, 124)]. LGBTQIA+ older adults are also often rendered invisible among service providers, healthcare practitioners, organizations, and policies (119) or explicitly targeted for exclusion (125, 126) that hinders efforts to build social support and connections.

#### Power inequities

Heteronormative cultural norms about family (cultural domain of power) are embedded in public policies and aging services (structural domain of power) about who is considered eligible for caregiver benefits that could reduce social isolation and loneliness among LGBTQIA+ older adults (e.g., families of choice). Disparities in benefits also serve to coerce LGBTQIA+ older adults into caregiving arrangements that may be less beneficial for their health and wellbeing (e.g., paid care by strangers) because that is all that is available and/or affordable (disciplinary domain). Based on past experiences of discrimination, LGBTQIA+ older adults may fear discrimination (intrapersonal domain) by strangers and be less receptive to or opt not to receive social or health services that could minimize loneliness or social isolation (interpersonal domain). All five of these domains of power interact in complex ways to produce inequitable outcomes for LGBTQIA+ older adults experiencing loneliness or social isolation, particularly in the social and community context (but also in health care access and quality) of the social determinants of health framework.

#### Proposed intervention: LInC: LGBTQIA+ intergenerational connections

Emerging research suggests that intergenerational support among LGBTQIA+ families of choice present promising opportunities for building connections that could reduce social and health disparities for LGBTQIA+ older adults [e.g., (115, 118)]. This case example presents LInC, a 12-week program that builds intergenerational connections and support among LGBTQIA+ communities. It also draws from research underscoring the benefits of arts-based programming to facilitate safe and supportive spaces for LGBTQIA+ communities to confront challenging experiences, share stories, and build networks of support [e.g., (127–129)]. Through LInC, LGBTQIA+ adults 18 years and older attend weekly workshops where they begin with a 30-min small group facilitated discussion (with trained facilitators) that incrementally delves into more complex issues relating to LGBTQIA+ lived experiences each week. The group then collectively participates in a creative activity that changes every week. The facilitator collaborates with a local LGBTQIA+ artist to channel the small group conversation into collaborative art that fosters self-expression, reflection, and community support. Participants spend 30 min learning basic techniques of that creative activity (e.g., painting with pastels, creative writing, photography, charcoal drawing, stage performance, crocheting, scrapbooking, podcasts) as it pertains to making one item. The diversity of creative activities allows participants with different skills, life experiences, and (dis)abilities to contribute in various ways each week. Participants subsequently break into teams with different skill levels and ages to collaborate on creating / producing something through that creative activity. The group is presented with several ideas on what to produce but can also decide on something different to create. At the end of the 12-week program, participants showcase their art in an exhibit (that could be internal to the group or external for the local community) to further spark conversation and connections.

**Figure 7:**
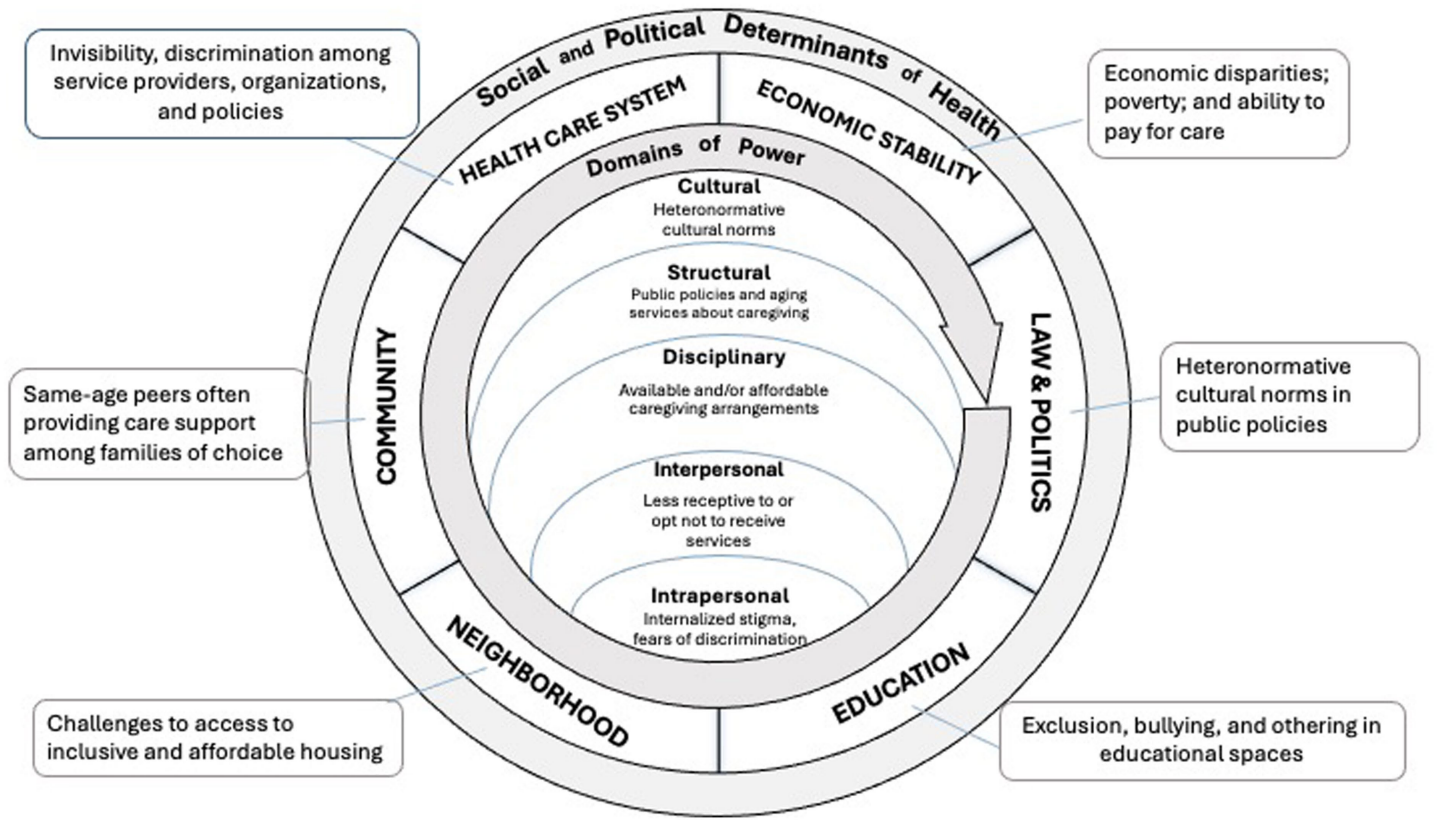
Intergenerational arts program (LInC) for LGBTQ+ older adults in the United States.

This intervention aims to build social connections and reduce loneliness among LGBTQIA+ communities, especially among older adults, by drawing on psychosocial theories of social justice [e.g., (130, 131)] that focus on the importance of social relationships among individuals and communities. Ultimately, LInC seeks to address inequities in power and disparities in social and political determinants of health by building opportunities for social connections through art. See Figure 7.

***Cultural Domain***: LInC counteracts cultural narratives that define LGBTQIA+ lived experiences solely through struggle, instead fostering opportunities to collectively experience and share joy. It also creates space to develop new cultural frames for lived experiences by facilitating intergenerational discussions within LGBTQIA+ communities.

***Structural Domain***: This intervention allows participants to collectively process structural barriers they have encountered as LGBTQIA+ community members and share strategies of survival and thriving through art.

***Disciplinary Domain***: This program also disrupts disciplinary norms around funding that implicitly drive the types of interventions nonprofits often are able to provide—specifically funding priorities that tend to favor traditional health-based interventions [e.g., (132)]—by creating a funded program that employs an arts-based intervention to address social isolation and loneliness.

***Interpersonal Domain***: By creating a program that employs diverse artistic mediums, LInC incorporates inclusive opportunities for participants with various abilities (and physical limitations) to create meaningful connections with each other. It also disrupts who is the “expert” of each creative endeavor by providing a wide range of art that draws on diverse knowledge from different backgrounds and lived experiences.

***Intrapersonal Domain***: LInC provides creative opportunities for participants to build a positive sense of self and assurance that others are interested in connecting with them.

## Conclusion

Concerns about social isolation and loneliness among older adults (and discussions about potential interventions) have only grown since the COVID-19 global pandemic [e.g., (6)], and research has well-documented disparities in health outcomes for older adults experiencing social isolation and loneliness [e.g., (7, 8, 13, 14, 16)]. This article presents a conceptual framework that bridges theories of power and social and political determinants of health to provide a new vision for developing interventions that address these inequities. The Equitable Aging in Health Conceptual Framework foregrounds power as a core driver of social and political determinants of health and the interventions that address health disparities that flow from inequities in power. Interventions that infuse justice must consider how these domains of power have shaped inequities in the past, present, and into the future–and across the life course–to achieve equitable health outcomes for older adults.

As illustrated in the six case examples above, the Equitable Aging in Health Conceptual Framework has application in a variety of contexts to address social isolation and loneliness for older adults. The first case example presents an intervention in community-based services to address social isolation among older rural–urban migrants in mainland China. The second case example presents an intervention at the cultural level that infuses Indigenous knowledge and experience into health care to address social isolation and loneliness among Taiwanese Indigenous communities. The third case example presents an intervention at the policy-level to address social isolation and loneliness among nursing residents in Spain. The fourth case example proposes cross-cultural legal clinics to address social isolation among non-European older migrants in Sweden. The fifth case example presents Ludotecas, a grassroots initiative to combat loneliness among older adults in Mexico. The sixth case example proposes LInC, an intergenerational arts-based program to build social connections and reduce loneliness among LGBTQIA+ older adults. By explicitly addressing power inequities and justice, these proposed interventions present a new paradigm for researchers, practitioners, and policymakers to reimagine ways to address inequities in social isolation and loneliness among older adults.

## Data Availability

The original contributions presented in the study are included in the article/supplementary material, further inquiries can be directed to the corresponding author.

## Footnotes

^1^Some scholars distinguish social drivers of health as the structural and systemic drivers of social determinants of health [e.g., (133)]. For purposes of this paper, we follow the World Health Organization’s framework, which incorporates structure and systems into social determinants of health by distinguishing structural from intermediate social determinants of health.
